# A Case Series on Penile Garroting: Causes, Considerations, and Consequences

**DOI:** 10.7759/cureus.70190

**Published:** 2024-09-25

**Authors:** Pradhyumna Koushik, Benjith Daniel, Vivek Meyyappan, Hariharasudhan Sekar, Sriram Krishnamoorthy

**Affiliations:** 1 Urology, Sri Ramachandra Institute of Higher Education and Research, Chennai, IND

**Keywords:** garroting, grading, injury, management, penis

## Abstract

Penile injuries from self-inflicted strangulation or garroting are uncommon. Children are more likely than young adults or elderly patients to sustain such injuries. Numerous foreign things, including hair, thread, rubber bands, metallic rings, nuts, and vacuum erection devices, have been reported to be used for garroting in the literature search. Determining the precise cause of such anomalous behavior can be difficult. If there is an underlying mental disorder, the situation becomes even more complex. While such mishaps in the elderly population may be for the relief of lower urinary tract symptoms such as frequency or incontinence, in young persons, they may be for sexual stimulation and erection augmentation. These devices tightly constrict the penis root, obstructing the venous return and causing corpora congestion that leads to ischemia, gangrene, or necrosis. We describe our observations of three incidents of penile garroting and its aftereffects. Here, we also talk about the several gadgets that our patients use and the methods that we employ to remove the constriction.

## Introduction

Self-inflicted strangulating (garroting) injuries to the penis are rare. Such injuries are more often seen in children and less commonly in young adults/geriatric patients. The literature search has documented the use of various foreign objects such as hair, thread, rubber bands, metallic rings, nuts, and vacuum erection devices for garroting [1-2]. Identifying the exact reason behind such unusual activities can be challenging. It becomes even more complex if there is any underlying psychiatric illness. While in young adults, it may be for sexual stimulation and enhancement of erection. In some Asian countries, it is a common practice amongst children or elderly patients to tie elastic rubber bands onto the shaft of their penis, aiming to get relief from intense storage symptoms like frequency, urgency, and/or incontinence [3]. Such devices snugly encircle the root of the penis, impede the venous return, and cause congestion of the corpora, resulting in penile tumescence. Persistence of venous congestion may lead to bothersome engorgement of the penis. Obstruction to arterial, venous, or lymphatic drainage of the penis may result in ischemia, gangrene, or necrosis. These constriction devices may also lead to mechanical injuries, including ulceration of penile skin, urethral injuries, constriction of the corpus cavernosa and corpus spongiosum, urethra-cutaneous fistula, and penile paresthesia [4-5]. An early diagnosis, rapid intervention, and treatment of underlying psychological abnormalities are the keys to success in most cases. In this manuscript, we report our experience of three cases of penile garroting and its sequelae. The various devices used by our patients and the techniques used to release the constriction are also discussed here. The purpose of this report is to emphasize the growing need to diagnose this potentially reversible condition early. This report also highlights the importance of a holistic approach in evaluating these patients, as many of them might have some underlying psychological or psychiatric illnesses, which, unless treated, may recur.

## Case presentation

Informed consents with signatures were obtained from all three patients, explaining and reassuring them that none of their identities would be revealed anywhere, and they were also ensured that their privacy would be protected by all means.

Case 1

An elderly gentleman in his early 90’s presented with the diagnosis of a non-healing penile ulcer. The ulcer was present around the coronal sulcus for 12 days.

**Figure 1:**
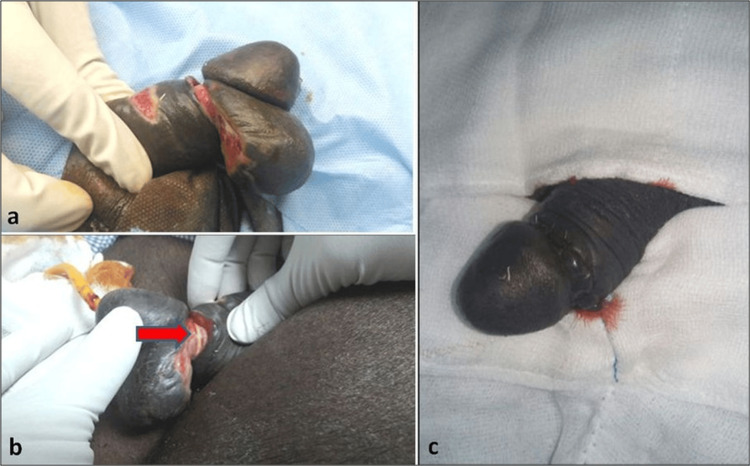
(a, b) Paraphimosis with a tight pink-colored constricting rubber band (red arrow) at the penile shaft; (c) post circumcision.

On examination, there was paraphimosis with a tight, pink-colored rubber band applied around the penile shaft proximal to the corona, deeply buried and causing penile skin erosion. The corpora and the urethra were found to be intact. It was later identified that the band was self-applied two weeks ago for management of urge urinary incontinence (Figure 1).

Case 2

**Figure 2:**
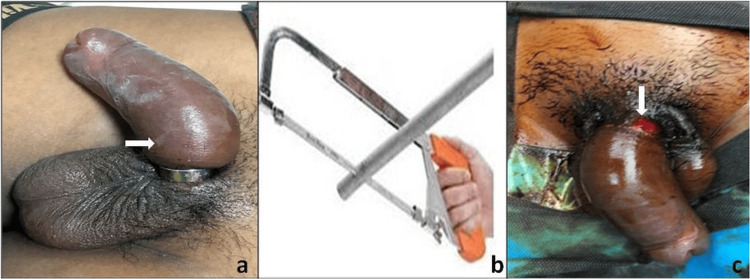
(a) Grossly edematous penis with constricting steel ring at the base of the penis; (b) Hacksaw ridge; (c) skin abrasion at the base of the penis after removal of the ring.

A teenage boy presented to the emergency room with a progressive swelling over the penis. He had applied a metal ring over the penis and had forgotten to remove the same. He had playfully applied the ring 12 hours ago but could not remove it thereafter. He did not have any difficulty in voiding. On local examination, a constricting stainless-steel ring was found encircling the root of the penis. Physical examination revealed a grossly edematous penis with hyperesthesia and warm penile skin (Figure 2).

Case 3 

**Figure 3:**
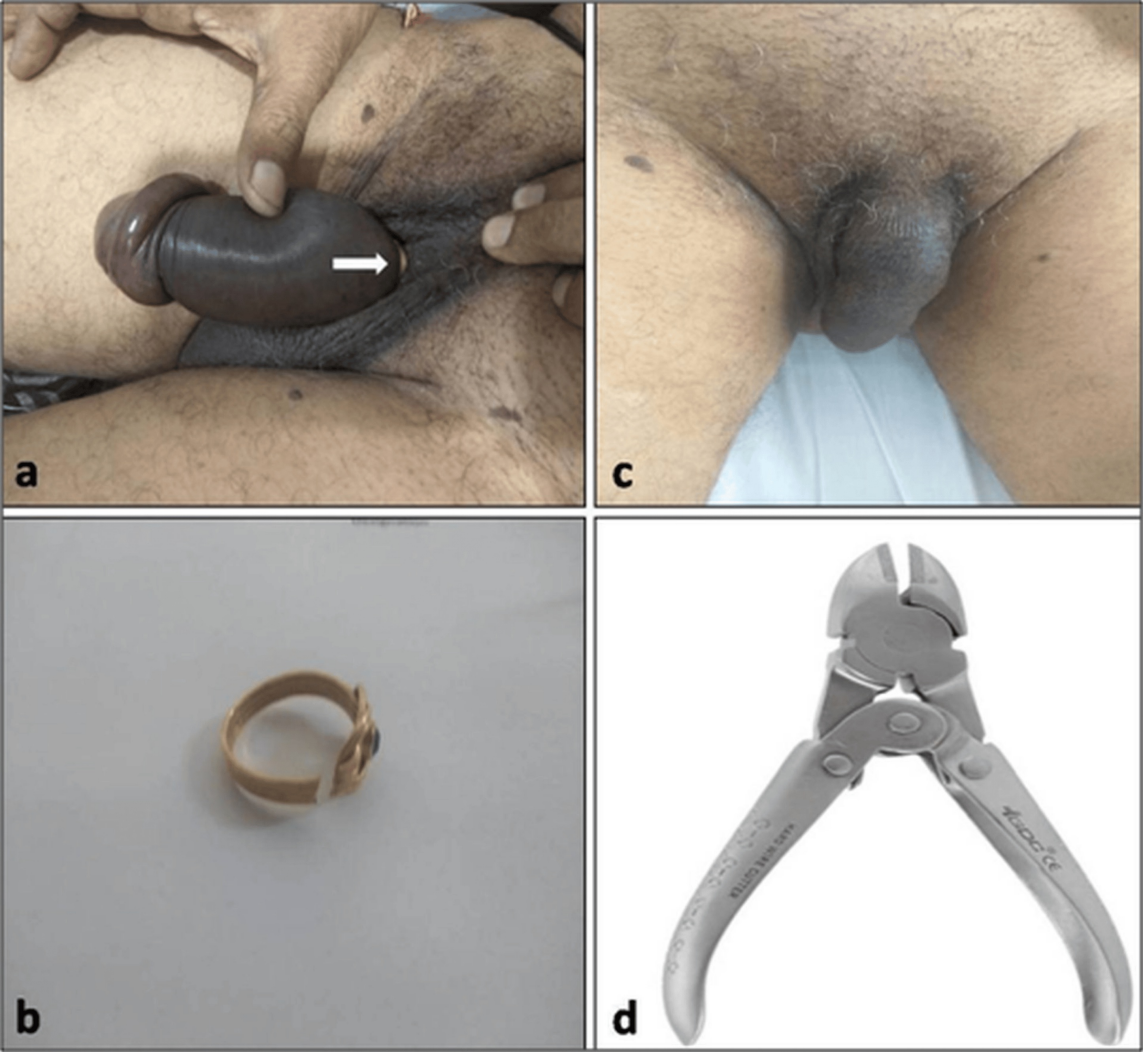
(a) Gold ring at the base of the penis causing penile oedema; (B) cut ring; (c) penis after removal of the ring; (d) rib cutter.

A gentleman in his 40's presented to the OPD with difficulty in voiding for 10 hours. He had applied his wedding ring to the penile shaft for sexual gratification. He gives a history of multiple such applications in the past and has had no difficulty removing them all these years. On examination, a gold ring with an external diameter of 2.5 cm was found at the root of the penis, causing strangulation and penile oedema (Figure 3).

The extent of damage was graded based on Bhat’s classification and compared with the revised grading system of Bashir et al. and Silberstein et al. [6-8].

Tables 1-2 describe the grading of penile garroting by Bhat et al. and Bashir et al., respectively [6,7]. Once the grading was done, all patients had been catheterized with an indwelling Foley catheter. The technique used to release the garrote was decided based on the material used, the grade of injury, and the duration of garroting. Table 3 summarizes the details of the presentation and management of all three cases in our study.

**Table 1:** The grading of penile injury by self-inflicted constriction bands. Bhat et al. [7]

Grade	Injuries
1	Distal penile oedema. No skin ulceration or urethral injury.
2	Distal oedema, skin and urethral trauma, corpus spongiosum compression and decreased penile sensation.
3	Skin and urethral trauma, no distal sensation.
4	Complete division, urethral fistula, corpus cavernosum compression, no distal sensation.
5	Gangrene, necrosis, or complete amputation of the distal penis.

**Table 2:** The grading of penile injury by self-inflicted constriction bands. Bashir and El-Barbary [6].

Grade of injury	The extent of damage caused
0	Constriction of skin without urethral injury
1	Partial division of corpus spongiosum with urethrocutaneous fistula
2	Complete division of corpus spongiosum and constriction of corpus cavernosum
3	Gangrene, necrosis, and amputation of the glans.

**Table 3:** Summary of presentation and management of all three cases.

No	Age (yrs)	Mental status	Type of constricting agent	Indications for insertion	Constriction site	Duration	Method of removal	Complication	Motive	Inflicted by
1	90	Normal	Rubber band	Urge leak	Distal shaft	2 weeks	Scissors	Wound infection	Self sexual gratification	Self
2	13	Normal	Stainless steel ring	Playful intent	Root	12 hours	Saw	Nil	Self sexual gratification	Self
3	47	Depression	Gold ring	Auto-erotism	Root	10 hours	Cutter	Nil	To prolong erection	Self

Table 4 summarizes the grading of penile garroting in our patients compared with Bhat et al., Bashir et al., and Silberstein et al. [6-8].

**Table 4:** Grading of penile garroting in our patients in comparison.

No	Features (on clinical examination)							Grading		
	Penile skin	Penile edema	Corpus spongiosum	Urethra	Cavernosum	Gangrene/necrosis	Amputation	Bhat	Bashir	Silberstein
1	3	3	3	2	3	3	3	3	0	Low
2	2	2	2	2	2	2	2	2	0	Low
3	1	1	1	1	1	1	1	1	0	Low

Outcome and follow-up

In Case 1, the rubber band was cut with scissors in the emergency room, and he was catheterized with a 16Fr Foley catheter. Later, he underwent circumcision (Figure 1c) and was discharged with a catheter, to be removed two days later. Follow-up after two months revealed the absence of a fistula or infection. The Mini Mental State Examination (MMSE) indicated a normal cognitive status. The patient was counseled regarding the prevention of such acts. A spontaneous decompression, excision of the ulcerated segment, and completion of circumcision were performed on our patient.

In Case 2, manual decompression and attempts to remove the constricting ring were unsuccessful. Under general anesthesia, various orthopedic instruments, including the drill, osteotome, K-wire cutter, and Gigli saw, were used to cut the ring but could not succeed. Finally, a plumbing metal saw (Figure 2b) was used to cut the metallic ring on the dorsal aspect, and the compression was relieved. The penile skin underneath the ring was found to be abraded (Figure 2c). The patient was catheterized with a 12Fr Foley catheter and was discharged on the second postoperative day after a successful voiding trial following catheter removal. The patient had a normal cognitive status on MMSE. On follow-up, the penis was normal with normal voiding. The patient underwent counseling and was educated regarding the hazards of such endeavors and advised to refrain from the same in the future.

In Case 3, the ring (Figure 3b) was cut using a cutter procured from the orthopedic department (Figure 3c), and the constriction was relieved (Figure 3d). The patient was on treatment for depression. He underwent psychiatric counseling a week later during the follow-up and was counseled and treated for his unusual sexual fetish.

Immediate release of the compressing agent was the prime motive for preserving the organ. The scissors, saw, or cutter were all the surgeon’s choices, with the sole priority of restoring vascularity to the organ. The decision-making was spontaneous, and the agent used to decompress was left to the surgeon’s discretion.

A proper mental state evaluation must be performed in all such patients. The underlying psychological factors need to be identified, and patients should be counseled accordingly. If any underlying psychiatric illness is identified, appropriate medical treatment needs to be initiated. Such measures would greatly help in preventing recurrences.

## Discussion

A garrote is a handheld weapon used to strangle an individual. This most often includes a wire, rope, metal chain, strings, or scarf. A continuous strangle of the penis would cause congestion, ischemia, and gangrene. The primary action should be to ensure an immediate release of the garroting body and minimize the extent of the damage.

The earliest reports of penile garrotting (as quoted by Trivedi et al. and Latabi et al.) are available from the publications of Gauthier as early as 1755 [9-10].

Constriction devices placed externally to compress the penis result in venous engorgement with an increase in the length and width of the penis. Such extraneous compressions obstruct the superficial veins and lymphatics, causing penile and preputial skin edema. The thicker Bucks fascia protects the cavernosal arteries from getting compressed and prevents corporal damage. However, a persistent garrotte would eventually obstruct arterial flow, resulting in ischemia, infarction, and penile gangrene. Broderick et al., in their penile blood flow study after vacuum-induced penile erections, observed that a constriction device kept for more than 30 minutes jeopardized penile blood flow [11]. Tanabe et al. report lymphoedema mimicking elephantiasis following prolonged garroting [12]. A protracted placement of these devices can lead to disastrous sequelae like urethro-cutaneous fistula, penile skin necrosis, fibrosis, ischemia to the urethra, and penile gangrene. A thorough evaluation with a detailed history and physical examination, including questions related to voiding dysfunction and the mental state, plays a pivotal role in the follow-up of these patients. Jandou et al. reinforce the need for a detailed mental assessment in such cases. They reported two similar cases in schizophrenic patients [13]. In one of the largest series of penile self-amputations, Greilsheimer and Groves observed that 87% of their patients had an underlying psychotic illness and concluded that schizophrenia was the most frequent mental abnormality associated with such patients [14].

Various authors have attempted grading penile strangulation. Bashir and El-Barbary describe four grades of penile constriction bands [6]. Bhat et al. proposed another grading system for penile injury [7]. Bhat’s classification gives a comprehensive grading system that considers all penile parameters and clearly distinguishes the various grades depending on the extent of damage with not much ambiguity. However, Bashir’s grading system is widely preferred owing to its simplicity and ease of clinical application.

Silberstein et al. proposed a simplified grading system for penile garroting [8]. The grading proposed by Bhat et al. was further simplified into low- and high-grade injuries. Grades 1 to 3 of the Bhat classification are grouped as low-grade injuries, while grades 4 and 5 are classified as high-grade injuries. This simplification by Silberstein et al. is justified based on the fact that lower-grade injuries most often require only a release of the constricting agent, and no further intervention may be needed. The higher-grade injuries may need surgical intervention. Table 4 gives a comparison of the three grading systems for penile garroting, which aided in selecting the appropriate management.

Silberstein et al. reported a greater incidence of high-grade injuries when the presentation was after 72 hours (29.1%) as compared to those who presented within 72 hours. In our case series, two patients presented early, within 72 hours. One patient with a rubber band over the penis presented late. All our patients had low-grade injuries. The object used as a constricting device plays a major role in the severity of the penile injury. Silberstein et al. observed that metallic objects accounted for only 22% of high-grade penile injuries, while non-metallic constricting devices caused more than three-fourths of the higher-grade injuries.

A step-wise approach proposed by Puvvada et al. gives a comprehensive idea about the type of approach to be adopted based on the grade of injury and the type of agent used to garrote the penis [15]. Their three-tier approach makes it easier and more convenient to remove the garroting agent rapidly and effectively without causing much damage to the penis.

Prompt decompression of the constricted penis is the key step in treating patients with penile garroting. Any delay in decompression will have potential side effects, including permanent erectile dysfunction, scarring, or psychological repercussions. Various decompression techniques have been described in the literature: corporal aspiration modified string technique, cutting devices, and degloving techniques [16]. String techniques were very popular as the resource needs were the least. Vahasarja et al. introduced this technique in 1993 on two of his patients [17]. Noh et al. further modified this technique by adding a puncture of the glans penis, resulting in venous decompression [18]. This added decompression makes the detumescence much more rapid, sustained, and effective. This technique also facilitates an effective release of the ischaemic blood entrapped in the congested corpora. A modified string technique proposed by Sarkar et al. included a corporal aspiration to decompress the engorged corpora followed by a tight winding of a string underneath the strangulating ring, distal advancement of the ring followed by an unwinding of the tightened string [19].

Cognitive assessment using Mini-mental score evaluation plays an important role in managing these patients. Cognitive assessment was done in all our patients using the MMSE scoring. A systematic approach to mental health assessment is the basic prerequisite while treating these patients, as failure to do this assessment would deprive them of an opportunity to correct themselves, which indirectly would lead to recurrences in the future. All our patients had normal cognitive status on evaluation, and one of them was on anti-depressants. Mandal et al. [20] suggested a strong correlation between psychological disorders and penile strangulation, while Jandou et al. [13] documented a strong possibility of underlying psychiatric disorders in such patients. Follow-up of these patients with psychiatric evaluation is of utmost importance, with educating the patients against such practices playing an important role. All our patients were counseled by psychiatrists with a focus on preventing such practices in the future. Follow-up is challenging in these patients owing to the nature of the condition and the embarrassment associated with it.

## Conclusions

Penile garroting by metallic and non-metallic objects is uncommon but warrants prompt intervention. Prompt release of constriction and restoration of blood supply is key to effective management. Associated urethral injury, though uncommon, should be ruled out in each patient. A mental status evaluation is mandatory in all such patients. It is prudent to use appropriate gadgets to ensure that the constriction is relieved without any damage to the organ.
